# Moving online: roadmap and long-term forecast

**DOI:** 10.1093/af/vfaa027

**Published:** 2020-07-23

**Authors:** John Scott Radcliffe, Debra K Aaron, Jodi Sterle, Marina A G von Keyserlingk, Nancy Irlbeck, Martin Maquivar, Meghan Wulster-Radcliffe, Cassandra Jones

**Affiliations:** 1 Purdue University, Department of Animal Science, West Lafayette, IN; 2 University of Kentucky, Department of Animal and Food Sciences, Lexington, KY; 3 Iowa State University, Department of Animal Science, Ames, IA; 4 Department of Land and Food Systems, The University of British Columbia, Animal Welfare Program, Faculty of Land and Food Services, Vancouver, BC, Canada; 5 Washington State University, Department of Animal Sciences, Pullman, WA; 6 American Society of Animal Science, Champaign, IL; 7 Department of Animal Sciences and Industry, Kansas State University, Animal Sciences and Industry, Manhattan, KS

**Keywords:** animal science, online learning, virtual learning

**Implications**

- Online teaching has been part of animal science departments for more than a decade, but COVID-19 hastened the conversion of many classes to fully virtual experiences.
- A virtual classroom will never fully replace the hands-on experiences associated with animal science courses, but the technology enabling online education is advancing.
- In the future, an estimated 90% of classes will have an online or virtual component.

## Introduction

Future concepts of university education have long incorporated movement to full virtual delivery but have met with skepticism citing the importance of a traditional college experience. In fact, the University of Phoenix and Purdue Global administer full undergraduate degree programs online, but the question always remains, is an online education as good as a traditional education (Ubell, 2019)? Is this truly what the future holds for university education? Animal science departments in particular have balked at making a complete shift to virtual learning, because of the importance in “hands on” experience in our field. Before the COVID-19 pandemic, campuses and animal science departments were evolving toward a digitized environment: transferring grade books to Learning Management Systems (LMS), moving from paper to online textbooks, providing recordings of lectures, offering online review sessions, using plagiarism detection software, providing the ability to register virtually and much more. The COVID-19 pandemic vastly accelerated the move to virtual classrooms and provided the first glimpse of what a fully virtual online animal science education might look like. Will the stop-gap procedures put in place during the recent COVID-19 pandemic truly influence the future of higher education?

There are over 4,000 public and private colleges and universities in the United States (https://www2.ed.gov/rschstat/catalog/colleges-universities.html). The U.S. postsecondary education system instructs more than 20 million students per year and generates more than $700 billion in revenue (https://nces.ed.gov/programs/coe/indicator_cud.asp). Current predictions are that COVID-19 pandemic will result in a 15% decrease in the number of U.S. students enrolled in a university and a 25% decrease in the number of international students enrolled at U.S. institutions (Smalley, 2020). Additionally, some universities are questioning whether students will be able to return to campus in the Fall of 2020. If virtual learning is the only delivery mechanism in the Fall of 2020, enrollments will decrease further (Korn et al., 2020). Should in-person classes resume in the Fall, concerns include adequate supplies of personal protective equipment, appropriate procedures for cleaning and disinfecting shared surfaces, requirements for health monitoring or testing for infection, infrastructure requirements needed to ensure social distancing and a changing student population. Fewer students may be on campus because of delayed admission decisions, canceled admissions tests (ACT, SAT), canceled campus visits, safety concerns of parents, or because students decide to stay closer to home during an uncertain period. Other students may find they prefer an online education where they can control their schedule and perhaps mitigate costs associated with a physical, on-campus presence. Regardless of the myriad of questions, courses, and classrooms are likely to be vastly different moving forward.

One of the pillars of animal science information transfer is face-to-face instruction and hands-on experiential learning activities. Thus, the COVID-19 pandemic challenged the most dedicated animal science educator. Most animal science departments have incorporated online education in their outreach programs and many animal scientists have been teaching online in a variety of formats for more than a decade. According to the National Center for Education Statistics (2018), before COVID-19, one third of all undergraduates were enrolled in online classes with 13% learning exclusively online (National Center for Education, 2018). Furthermore, online course enrollment increased for 14 consecutive years (Seaman et al., 2018). Because animal science departments at land-grant universities have closely aligned teaching and extension missions, many have long-term online distance learning capabilities.

With the need to move all education online within a short period of time, the dedicated faculty in the animal sciences made it work and learned in the process. Those lessons will allow teaching and outreach faculty to create tools to improve education and potentially reach people who do not have the resources to attend a university campus. This paper is the result of discussion of many educators in the animal sciences who came together to discuss the challenges and lessons learned during the COVID-19 pandemic. Readers may also view the webinar that provided the foundation for this paper (Radcliffe et al., 2020; https://vimeo.com/403860309). These experiences are likely to help reshape the global landscape of higher education and may serve as a roadmap for how educators engage in both online and in-person education in the future. The aims of this article are to discuss methodologies to facilitate the shift to online instruction and examine some philosophies associated with online education.

## Moving Online: The Virtual Push Today and in the Future!

**Figure 1.**
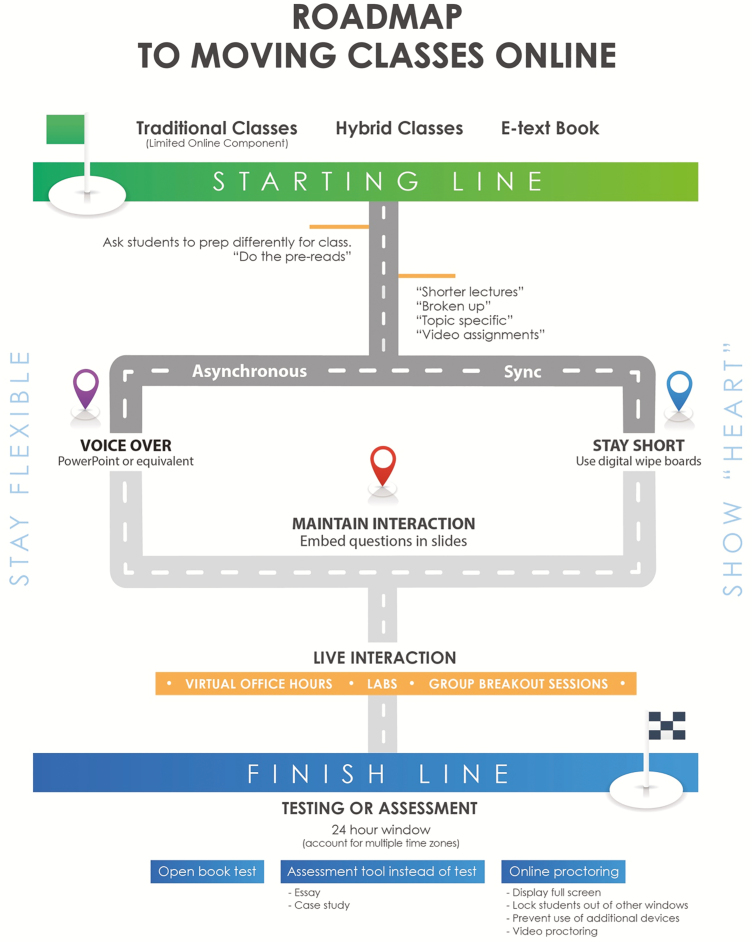
Roadmap to moving classes online.

Some educators had 2 weeks to move to online instruction, which for some began with a hybrid version of a course (in-class and online instruction). Others had 72 hours to move teaching from face-to-face to 100% online. As a result, educators across the world have become experienced in rapidly moving courses to an online platform—not necessarily experts in best practices, but certainly experienced in “getting it done.” Figure 1 is a roadmap that describes the collective experiences of the authors as guidance for moving classes online.

**Figure 2.**
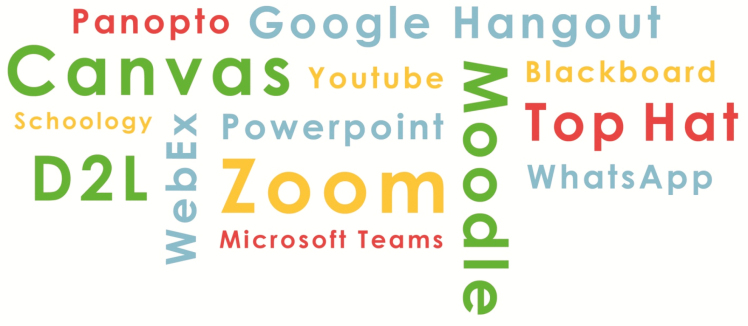
Many of the common tools used for online teaching today. Note that the authors do not endorse or recommend a specific product or tool. Costs, advantages, and disadvantages are variable with each product.

As depicted in Figure 1, the path taken largely depends on the type of online classroom an educator chooses. There are many types of virtual delivery including: Synchronous Delivery, Asynchronous Delivery, Hybrid Delivery, and Online Laboratory. There is an abundance of research on the pros and cons of teaching synchronously and asynchronously (Bernard et al., 2009; Jordan et al., 2013; Pei and Wu, 2019) and a number of tools currently available to facilitate building of an online classroom (Figure 2).

- Synchronous Delivery: Traditional classroom delivery occurs when instructors interact with students during designated times on specific days. Video conferencing technologies such as Zoom, WebEx, or Microsoft Teams, for example, allow educators to maintain this model online. This format requires that each student have a computer or cell phone and a connection to the internet at the designated time. Synchronous delivery allows instant student engagement and allows instructors to pace course content and depth based on student reaction in real-time. The chat and “raise hand” functions of many of these technologies enable students to ask questions in a low-risk manner, and instructors can respond live and alter remaining class content as needed. Synchronous delivery in a virtual learning environment is not without distractions. There is, just as when attending a face-to-face lecture, an expectation of the students to listen and engage with the material. As with in-person classes, there are always students who are involved on their computers or cell phones in ways that are not part of the class. Moreover, the engaged student may have added challenges that come with a home office (or other makeshift learning environment). Another possible requirement of synchronous delivery of virtual content is availability of high-speed internet at designated times.
- Asynchronous Delivery: Courses with asynchronous delivery rely on LMS (virtual platforms that help faculty interact with students on and off campus), e-mails, and discussion boards that let students complete course requirements on their own time. The primary advantage of asynchronous delivery for both educators and students is the flexibility that comes with this type of delivery. Access to the course is available when it is convenient for both parties. Depending on the course, asynchronous delivery may also allow students to alter the pace of their learning. Students can move quickly through material that they have mastered in other courses or slow down to listen to segments of lectures that were confusing or more difficult to comprehend. These advantages are also the greatest disadvantages of asynchronous delivery. Not all students excel in a self-guided learning environment; many need the structure of a classroom environment and schedule to succeed. It may also be more difficult for instructors to identify if students are struggling with a concept. Asynchronous delivery also challenges educators to find novel ways to promote thoughtful discussion, a necessary component of critical thinking and learning.
- Hybrid Delivery: To capture the student engagement advantage offered by synchronous learning with the flexibility of asynchronous delivery, many animal science instructors have adopted a hybrid model. This model uses asynchronous delivery of some portions of course content, such as lectures and assignments. Other portions, such class “meet-up” sessions are available at designated times to provide touchpoints for instructors and students. Recorded sessions are available for students who did not attend at the designated time. It also allows students to access and review content that was delivered live.
- Online Laboratory Classrooms: Laboratory classes are far more difficult to move online compared with the lectures. Most animal science departments take pride in their experiential learning activities, which are not easily replaceable with online learning exercises. However, requirements to decrease transmission of COVID-19 necessitated the move of laboratory classes online. Ease of transition to an online format is highly dependent on laboratory subject matter. An applied nutrition lab focused on diet formulation or a math-based genetics lab may be much easier to deliver online compared with introductory animal science or animal production laboratories, which traditionally have significant components of hands-on learning. Virtual tours of farms or detailed videos showing dissection of a rumen, for example, have been used as replacement exercises. To keep students engaged, educators should make the online laboratory as interactive as possible (Radcliffe, 2020). For instance, limiting video clips to short segments creates a natural break where students can engage through questions and discussion prior to moving on (https://www.purdue.edu/innovativelearning/teaching-remotely/).

The success of online instruction for tasks or concepts more easily mastered using hands-on techniques is relatively unknown. In a recent study, first- and second-year veterinary students learned online to administer a corneal nerve block (CNB) in dairy calves prior to dehorning. Although those students who learned online were just as effective in administering the CNB compared with the group that received hands-on training, they were less confident and had poorer technical skills (Winder et al., 2017). The authors go on to state that “*while online training is not recommended as a sole method of instruction, in the absence of available hands-on training it may be a suitable alternative method.*” Technology will no doubt become increasingly important in online laboratory experiences as state-of-the-art 3-D interactive modules and/or simulators become available.

Most animal science faculty moved quickly to asynchronous delivery of classes because it was the easiest and fastest method to adopt, and arguably the most convenient for students. However, as COVID-19 quickly drives the development of new technology for online education and pushes educators to learn how to use the available online technologies (Figure 2) increased adoption of synchronous active online classrooms is likely (Reimers and Schleicher, 2020).

Teaching, like any skill, takes time and practice to develop. Effectively teaching in a virtual environment will also take time to develop. For the most part, universities have allowed professors to decide if or when they would like to develop this skill set but studies show that before COVID-19 only 9% of instructors preferred online to in-person teaching (Pomerantz and Brooks, 2017). COVID-19 will require almost all teachers to develop this skill set. How does the virtual classroom affect faculty that cannot adapt to the changes? Will retirements and resignations of talented teaching faculty be more commonplace? Will there be long-term changes in who is attracted to teach at a university?

Additionally, while there has been a great deal of emphasis on learning to teach online, the flip side is will students be able to learn virtually? Some students will adapt quickly, and some will not. Research demonstrates that marginal students will have the most difficult time adapting to online learning (Xu and Jaggars, 2014). These results, coupled with changes in learning system availability, will inadvertently shift student populations.

**Figure 3.**
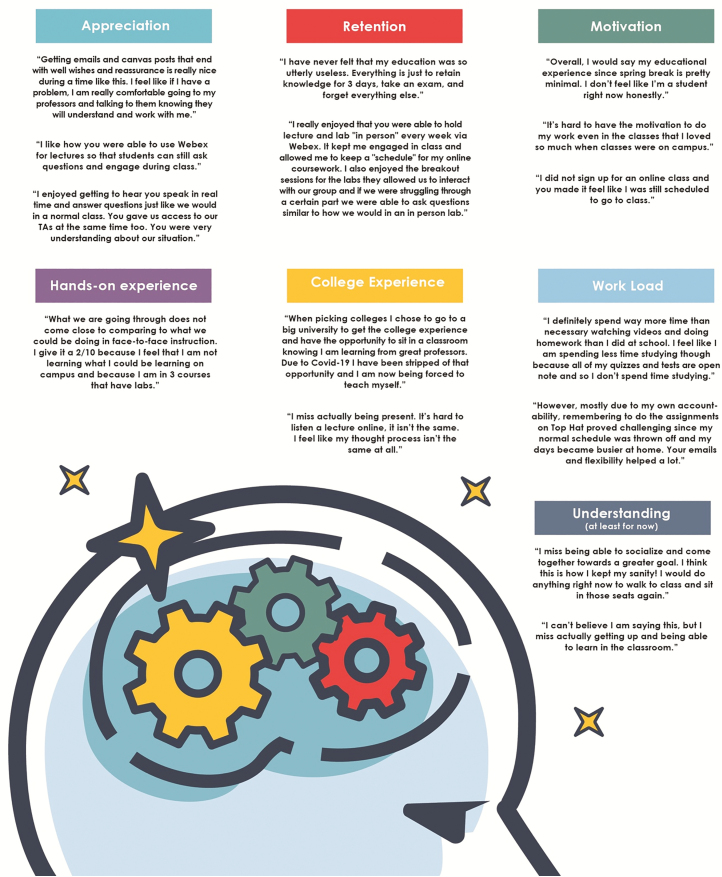
Informal student responses.

To gauge how students were coping with the sudden switch to online instruction, an online assignment was given to students in Dr Jodi Sterle’s ANS 211: Issues Facing Animal Science course at Iowa State University and in Dr Scott Radcliffe’s ANS 324: Applied Nutrition course at Purdue University. Students were asked to submit responses to only the questions they felt comfortable answering. Only a “complete/incomplete” grade was given, and as long as a student answered something, it was considered “complete.” Students in Dr Sterle’s class are mostly sophomores, and most, but not all, were Animal Science and Dairy Science majors. The students in Dr Radcliffe’s class were mostly juniors and seniors and most were Animal Science majors. This exercise was meant to be only a check-in to see how students were doing and an opportunity to reflect on their current experiences. Students were very honest and forthcoming, as well as compassionate and appreciative of their instructors’ efforts to move to online quickly. Seven main themes emerged (Appreciation, Hands-on Learning, Retention, College Experience, Motivation, Workload, and Understanding) and these themes are depicted in Figure 3. Not necessarily denoted in Figure 3 was the overwhelming sentiment from Dr Sterle’s students that they retained less material and the sentiment from Dr Radcliffe’s class that the course was heavily dependent on online technologies before the shift helped.

## Testing and Assessment

Assessment of a student’s understanding of the subject matter has been one of the biggest challenges as educators moved to online courses because students are unsupervised and have unlimited access to the internet. Additionally, educational scientists have spent time studying best practices for online assessment (Diamadis and Polyzos, 2005), but in the rapid movement associated with COVID-19 few educators spent significant time evaluating their options. Online testing will continue to be a challenging area moving into the future. However, there are several options that are continuously evolving:

- Integrity pledges: Many universities already have or are adopting some form of academic integrity pledge that the students must sign prior to taking an exam.
- Test alternatives: Assessments of what a student “knows” may also look different in the future compared with the standard written exam. Educators will adopt alternative approaches to assess a student’s knowledge where appropriate. Some examples might be a written “essay” assignment, preparation of a video summarizing a concept, or an online oral presentation.
- Open-book exams: In this case, instructors will accept that students have access to the material they would previously have had to memorize and open-book exams that concentrate on application of information will be used. When objectives are clear, open-book exams can be more challenging for students than traditional closed-book exams (Green et al., 2016).
- Online testing and proctoring tools: Proctoring systems have the capability to fully display an exam on a computer monitor and prevent the opening of additional windows. Students are locked out of an exam if they try to open another window on their computer. Some systems even prevent the use of other devices registered to the same student. More advanced systems offer online video proctoring if so desired. Online proctoring systems tend to be successful at reducing cheating and assuring student compliance but have been met with skepticism by students who view them as intrusive, potentially discriminatory and an additional cost (Dimeo, 2017).

Adequate testing and assessment techniques have been difficult to implement in the switch to an online classroom. However, future online and virtual testing capabilities that are both flexible and secure are likely to be incorporated into virtual and traditionally taught classes.

## Advising and Mentoring

Many universities were already offering online meetings as an option for meeting with advisees, while others quickly migrated to online meetings. Advising challenges include scheduling meetings, reminding students about meetings, and keeping students. The online meeting platform must be Family Educational Rights and Privacy Act (FERPA) compliant and the advisor must be aware of data that can and cannot be shared.

The impact that animal science educators make on their students go beyond teaching various disciplines and extend to roles as advisors, mentors, and on some levels, as confidantes. In the procession towards digital teaching platforms, it will be imperative to identify methods that maintain the critical interpersonal interactions that allow animal science educators to serve a multitude of roles.

## Equity and Inclusion in a Digital Age

Within the last decade, higher education has made a real and lasting effort to enhance equity and inclusion (Proctor, 2005; Anderson, 2019). A complete discussion on the potential switch to a virtual classroom today must address the issue of equity and inclusion in the virtual space. However, as none of the authors of this paper are experts in this area, they recognize that the equity and inclusion issues observed in the transition to virtual classrooms are important issues that must be discussed at the highest levels of the university system. One arbitrary grouping of students observed in animal science departments by these authors included 1) rural students, 2) students who left university and added additional jobs, 3) urban students, and 4) first-generation students. Interestingly, although these four groups represent different demographics, students struggled with the same issues: lack of access to a computer, lack of adequate internet access and/or a safe supportive learning environment, and additional responsibilities that reduced time available for studying.

**Figure 4.**
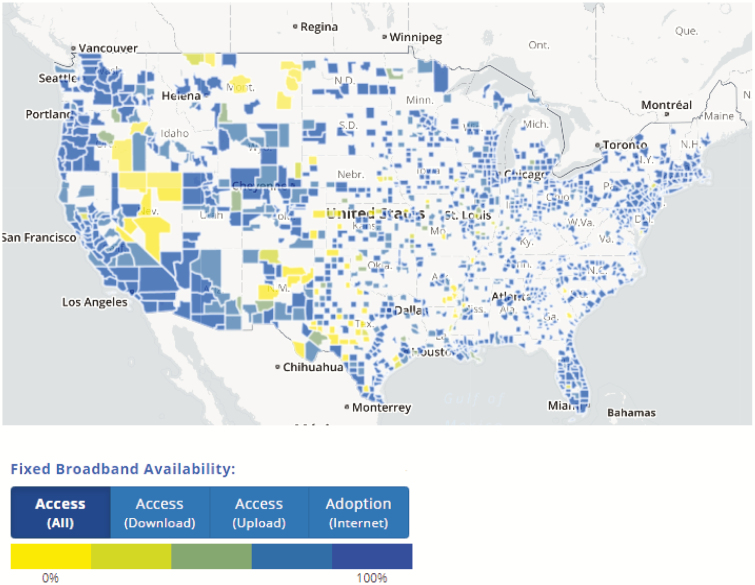
Availability of high-speed internet by county across the U.S. Federal Communications Commission (2017).

**Figure 5.**
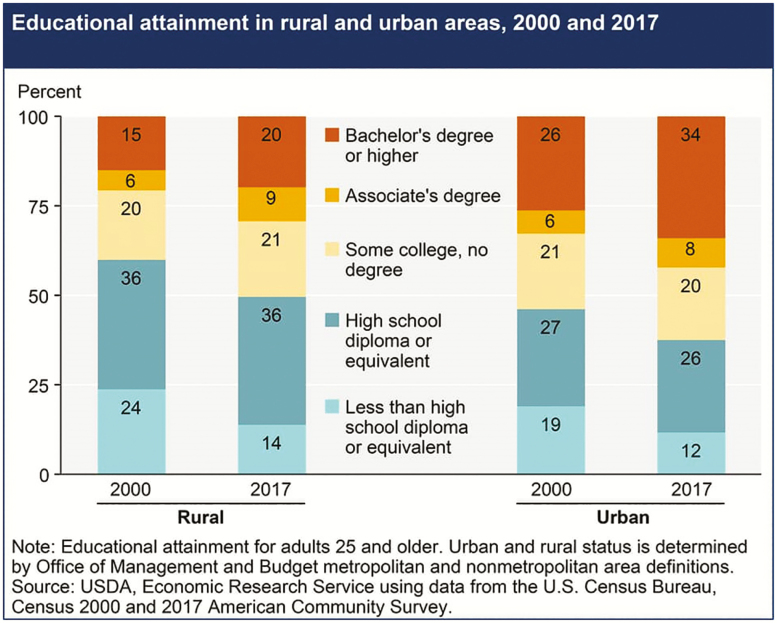
Educational attainment in rural and urban areas (Marré, 2017).

As an example, when the COVID-19 crisis dictated virtual classrooms, many students returned to family homes in the midst of spring calving or planting season. Suddenly, many students were working 8- to 12-hour days on the ranch or farm before beginning their studies for the day. Additionally, many students already struggle to balance part-time jobs with coursework when they are living on campus and have structured schedules. The self-discipline necessary to focus on college content may become even more difficult when living at a family home with potential work hours doubled or tripled. It is unrealistic to expect that a student will be able to focus only on their education in a society where social distancing, increasing unemployment, and agricultural supply chain issues will be the norm for the foreseeable future. Additionally, as the economic ramifications of the COVID-19 pandemic become clearer and students realize that education may be less expensive not living on campus, it is likely that many animal science students will elect to stay and learn while living at a family home versus return to “brick and mortar” university systems. The potential loss of this group of students must be addressed. The challenge of retaining these students is further exacerbated by the fact that even the most dedicated scholars may be limited by access to technology and high-speed internet when living on a farm or ranch in rural areas (Figure 4; https://www.fcc.gov/reports-research/maps/connect2health/#ll=41.22274,-96.269529&z=4&t=broadband&bbm=fixed_access&dmf=pop_urbanrural$0_50&zlt=county). Anecdotal reports by animal science faculty concerning students who left campus, indicate there are many people who download lectures or take exams in the parking lots of fast-food restaurants because of limited broadband speed in their own homes. Animal science educators must be cognizant of all these challenges so COVID-19 does not become the justification for widening the achievement gap between different student groups (Figure 5, Marré, 2017).

**Example 1: Movement of a Sophomore-Level Nutrition Class from Hybrid to Fully Online**

Dr Jones’ lecture-based, sophomore-level nutrition class was already using a hybrid model of instruction. Before the COVID-19 pandemic, Dr Jones delivered content live to students in a lecture hall on designated times and days but allowed students to access the class on Zoom. She started using this instruction protocol in Spring 2019, when several winter storms closed local schools but the University was not closed. Some students felt unsafe traveling to class on icy roads. Feedback revealed that many nontraditional students particularly appreciated the flexibility of having a Zoom link because they were traveling from a nearby military base or had young children. This year, Dr Jones also posted a video recording within 24 hours of a lecture to the class Learning Management System so students could watch lectures, if they missed class due to judging competitions, illness, or family/military obligations. She was surprised to find that having Zoom available live or as a recorded lecture had little impact on class attendance, but she recognizes that may be different for a class offered at 8:30 a.m. instead of 11:30 a.m. In response to the university movement to continuous virtual learning in March 2020, Dr Jones shifted her class to asynchronous delivery, but maintained a Wednesday “meet up” session for students that wanted to ask questions or go over specific material. After the first exam, when several students underestimated the studying needed to answer 30 questions in 60 min in an open-book, online exam, Dr Jones also began a live Kahoot session to help students better assess their mastery of material.

**Example 2: Movement of Research Methods in Applied Biology in Canada (Adapted with Permission from von Keyserlingk, 2020)**

At the beginning of the COVID-19 pandemic, Dr von Keyserlingk (University of British Columbia) moved a Research Methods in Applied Biology course fully online. Students who are thinking about whether to complete an undergraduate thesis in their final year or are simply interested in research take the course. The course is grounded in experiential learning. Students identify and collaborate with a scientist/researcher working in an area of the student’s interest. During the term, students volunteer a minimum of 20 hours with the scientist and work on a research project. Although most researchers are located on campus, over the past decade the virtual world has helped expand the areas in which students identified researchers as potential mentors. For instance, this past year one student was interested in working on biomechanics of horses and found a scientist working on this subject in the United Kingdom, while another student found someone studying penguins on an island off the coast of Argentina. In these cases, the students engaged in research through data entry tasks, scoring of videos, or other tasks needed by the researcher.

Much of this course required peer-to-peer review of the various required assignments (e.g., preparation of a poster summarizing some of the research done in their mentors’ laboratory). The breakout room tab on Zoom was a lifesaver as we were able to put students into small groups of 3 to 4 to allow for peer-review of their posters. Within each breakout room, students shared their screens one at a time and requested critical feedback from their colleagues. This was then followed up by each student presenting the final version of their poster a week later using Zoom—our attempt at providing the virtual equivalent of an undergraduate student poster session at a conference. In the voice of one of the students it was clearly a success: *“The online poster presentation was a rare and valuable opportunity to be treated as a scientific equal as an undergraduate*. *” (Student, APBI 398, April 2020)*

## Conclusions

Because of the rapid nature of the change to a virtual teaching landscape in response to the COVID-19 pandemic, this article focuses on the rapid move to virtual classrooms, leading to a more important question: What will animal science classrooms look like in the future?

- The virtual portions of animal science courses will be greatly enhanced. This was likely to occur with or without the COVID-19 pandemic, but the pace of this change will be increased.
- Animal science departments will recover from changes imposed by the COVID-19 pandemic with a greater understanding and appreciation of digital teaching tools. As a result, instructors will be able to spend time incorporating these tools into their classrooms in productive and complementary ways.
- Instructors of animal science courses will view face-to-face instruction as more valuable, utilizing online tools to enhance face-to-face interactions.
- Quality virtual classrooms may work better for some subjects and provide different opportunities for some students, resulting in creation of more courses that are 100% online and cater to those students.
- Some subjects and students may require movement of the virtual environment to a different space than currently envisioned.
- Collaboration across universities may be used more frequently to teach core online classes. Experts within a subject area may be brought together from different institutions, resulting in a course that is better than anything that could have been developed individually.

This article covers the changes that were made in the move to online instruction and the associated difficulties associated with those changes. While difficult, the challenges met and overcome represent extraordinary opportunities to animal science departments as they move to virtual environments.

## About the Authors

**John Scott Radcliffe** is Professor in the Department of Animal Sciences at Purdue University. He received his BS, MS, and PhD from Virginia Tech and is a professor at Purdue University. Dr Radcliffe’s research interests include environmental nutrition (how do we feed pigs to ensure the highest level of environmental sustainability?) and gut health. He teaches Applied Animal Nutrition and he developed an online textbook for this class. He teaches the class as a hybrid online course with a significant portion of synchronous lectures online or in person and works with students on delivery of group projects to enhance communication about animal nutrition.

**Debra K. Aaron** is a Professor and Director of Undergraduate Studies in Animal Sciences at the University of Kentucky. She teaches Introduction to Livestock and Poultry Production and Animal Breeding and Genetics. She has been faculty advisor to the UK Block and Bridle Club since 1992 and was national president, 2012–2014. Her awards include Advanced Degree Graduate of Distinction, Oklahoma State University Animal Sciences Department (2010); ASAS Fellow-Teaching (2012); ASAS Distinguished Teacher (2013); Distinguished Alumnus, UK Department of Animal and Food Sciences (2018); Teacher Who Made a Difference, UK College of Education (2020). She served as ASAS President , 2014–2015.

**Dr Jodi Sterle** is the Harman Endowed Professor in Undergraduate Teaching and Learning in the Department of Animal Science at Iowa State University. She serves as Undergraduate Teaching Coordinator for over 1000 students enrolled in Animal Science. Dr Sterle earned a BS in Animal Science from Michigan State University and a MS and PhD in Swine Reproductive Management from the University of Missouri. Dr Sterle teaches courses in introductory animal science, issues facing animal science, and The Art and Heritage of Livestock. She advises several student organizations, and serves as an academic adviser for 80 students.

**Dr Marina (Nina) von Keyserlingk** is Natural Sciences and Engineering Research Council Industrial Research Chair in Animal Welfare in the Faculty of Land and Food Systems, University of British Columbia. Her interdisciplinary approach has focused on identifying measures and ways of improving the lives of animals under our care. In addition to research she has a strong interest in teaching and in 2008 was awarded the Killam teaching award for her outstanding contributions to both graduate and undergraduate teaching. She is interested in finding ways of demystifying the world of research for undergraduates.

**Nancy A. Irlbeck** is currently on faculty at Washington State University in the Department of Animal Sciences as a Comparative Nutritionist. She is in a primarily teaching role as a Clinical Associate Professor after retiring from Colorado State University after 27 years. Dr Irlbeck holds Emeritus positions from CSU as an Associate Professor of Animal Sciences and as Associate Dean of Academic Affairs from the College of Agricultural Sciences. Dr Irlbeck has Bachelor of Science and Master of Science degrees in Animal Sciences from Iowa State University and a PhD in Ruminant Nutrition from the University of Nebraska-Lincoln.

**Dr Martin G. Maquivar** is a faculty member in the Department of Animal Sciences at Washington State University. Martin teaches Introduction to Animal Sciences and Reproductive Physiology. He supervises undergraduate research, study abroad programs, and works with Extension faculty conducting training in Spanish. His research interest is in ruminant reproduction and management. Martin is originally from Mexico City, Mexico, where he graduated from Veterinary Medicine School, and earned a master degree in nutrition and reproduction of ruminants at UNAM.

**Meghan Wulster-Radcliffe** is the CEO of the American Society of Animal Science (ASAS). She received an undergraduate degree from the University of Wisconsin-Madison and her MS and PhD from Virginia Tech. Meghan was a postdoctoral fellow at the USDA Sheep Experiment Station in Dubois, ID and at Purdue University. Before taking the position of CEO of ASAS, Meghan worked in academics, government, and industry and throughout those positions continued teaching Physiology. Meghan helped create the National Academic Quadrathalon finals and ensures adequate undergraduate programming at ASAS meetings.

**Dr Cassandra Jones** is Undergraduate Research Coordinator in the Department of Animal Sciences & Industry at Kansas State University. She coordinates undergraduate students interested in research projects across disciplines and species. Cassandra teaches Undergraduate Research in Animal Sciences & Industry and Fundamentals of Nutrition. Her research efforts include evaluating the effects of ingredients or feed processing technologies on feed safety and animal nutrition. She is formally trained as an applied swine nutritionist.
